# Pathogenic Nontuberculous Mycobacteria Resist and Inactivate Cathelicidin: Implication of a Novel Role for Polar Mycobacterial Lipids

**DOI:** 10.1371/journal.pone.0126994

**Published:** 2015-05-18

**Authors:** Jennifer R. Honda, Tamara Hess, Kenneth C. Malcolm, Alida R. Ovrutsky, Xiyuan Bai, Vida R. Irani, Karen M. Dobos, Edward D. Chan, Sonia C. Flores

**Affiliations:** 1 Department of Medicine, Division of Pulmonary Sciences and Critical Care Medicine, University of Colorado Anschutz Medical Campus, Aurora, Colorado, United States of America; 2 National Jewish Health, Denver, Colorado, United States of America; 3 Department of Microbiology, Immunology, and Pathology, Colorado State University, Fort Collins, Colorado, United States of America; 4 Department of Biology, Indiana University of Pennsylvania, Indiana, Pennsylvania, United States of America; 5 Denver Veterans Affairs Medical Center, Denver, Colorado, United States of America; Cambridge University, UNITED KINGDOM

## Abstract

Nontuberculous mycobacteria (NTM) are a large group of environmental organisms with worldwide distribution, but only a relatively few are known to be pathogenic. Chronic, debilitating lung disease is the most common manifestation of NTM infection, which is often refractory to treatment. The incidence and prevalence of NTM lung disease are increasing in the United States and in many parts of the world. Hence, a more complete understanding of NTM pathogenesis will provide the foundation to develop innovative approaches to treat this recalcitrant disease. Herein, we demonstrate that several species of NTM show broad resistance to the antimicrobial peptide, cathelicidin (LL-37). Resistance to LL-37 was not significantly different between *M*. *avium* that contain serovar-specific glycopeptidolipid (GPL, *M*. *avium* ^ssGPL^) and *M*. *avium* that do not (*M*. *avium* ^ΔssGPL^). Similarly, *M*. *abscessus* containing non-specific GPL (*M*. *abscessus* ^nsGPL(+)^) or lacking nsGPL (*M*. *abscessus* ^nsGPL(-)^) remained equally resistant to LL-37. These findings would support the notion that GPL are not the components responsible for NTM resistance to LL-37. Unexpectedly, the growth of *M*. *abscessus* ^nsGPL(-)^ increased with LL-37 or scrambled LL-37 peptide in a dose-dependent fashion. We also discovered that LL-37 exposed to NTM had reduced antimicrobial activity, and initial work indicates that this is likely due to inactivation of LL-37 by lipid component(s) of the NTM cell envelope. We conclude that pathogenic NTM resist and inactivate LL-37. The mechanism by which NTM circumvent the antimicrobial activity of LL-37 remains to be determined.

## Introduction

Nontuberculous mycobacteria (NTM) are aquaphilic and geophilic environmental organisms frequently recovered from potable and natural water sources, plumbing systems, and soil [1–3]. The majority of the more than 169 catalogued NTM species are not known to be pathogenic, but several, including *Mycobacterium avium*, *Mycobacterium intracellulare*, *Mycobacterium abscessus*, *Mycobacterium kansasii*, *Mycobacterium xenopi*, and *Mycobacterium malmoense* are clinically significant and of growing concern for several reasons [4]. First, the incidence and prevalence of NTM lung disease are on the rise [5–9]. Second, NTM pulmonary infections are expensive to treat, ranking second only to Legionnaires’ disease for inpatient treatment costs attributed to water-associated diseases [10]. Third, while the prevailing notion is that NTM are not contagious [11, 12], person-to-person transmission has been proposed [13]. Fourth, NTM lung diseases are often resistant to treatment despite the use of lengthy, multi-drug regimens [14].

The surface of most bacteria including the cell wall of NTM is negatively charged, allowing cationic antimicrobial peptides to bind and serve as first line, innate immune defenses against invading pathogens [15, 16]. The antimicrobial peptide cathelicidin (LL-37), binds, inserts, and forms pores in the negatively charged membrane of a variety of Gram-negative and Gram-positive bacteria [17–20]. LL-37 has also been shown to kill *Mycobacterium tuberculosis* (*Mtb)* [18, 21]. Despite its broad-spectrum of activity, LL-37 did not inhibit the growth of *M*. *avium hominissuis* [22, 23].

In the current study, we sought to examine whether other clinically relevant pathogenic species of NTM including *M*. *avium* Chester and *M*. *intracellulare* are resistant to LL-37. Since NTM cell envelope components have been implicated to play a role in immune evasion, they may also contribute to resistance against LL-37 [22]. Among the various cell wall components of NTM that have been characterized, glycopeptidolipids (GPL) are lipid molecules linked to the pathogenesis of NTM infections [24–30]. More specifically, GPL are unique to NTM and contribute to colony morphology, sliding motility, and biofilm formation [31–35]. NTM such as *M*. *avium* produce polar serovar-specific GPL (ssGPL) comprised of unique serovar-specific oligosaccharides covalently bound to a basic tripeptide amino acid alcohol core, fatty acid, methylated rhammose, and 6-deoxytalose [36–38]. Other NTM such as *M*. *abscessus* do not produce ssGPL, but rather express non-polar, diglycosylated non-specific GPL (nsGPL) [32]. Thus, we also investigated whether resistance to LL-37 in *M*. *avium* and *M*. *abscessus* strains is dependent on GPL. Finally, we determined whether the antimicrobial activity of LL-37 is compromised after incubation with NTM.

## Materials and Methods

### Bacteria

*Salmonella enteriditis* Uganda and *Salmonella non-typhi* Nairobi were kind gifts from Dr. Edward Janoff (University of Colorado Anschutz Medical Campus, Aurora, Colorado). *M*. *avium* Chester was obtained from ATCC (#700737) and *M*. *intracellulare* 9141 was kindly provided by Dr. Leonid Heifits (National Jewish Health) [39].

The original characterization of *M*. *avium* 920A6 serovar 8 wildtype and the allelic exchange mutagenesis of the rhamnosyltransferase (*rtfA*) gene to generate a null strain lacking ssGPL have been previously reported [40]. In this study, we refer to *M*. *avium* serovar 8 wildtype as *M*. *avium* ^ssGPL^ (containing ssGPL) and the ssGPL null strain as *M*. *avium* ^ΔssGPL^ (lacking ssGPL).

*M*. *abscessus* produce only nsGPL [32]. *M*. *abscessus* variants with nsGPL (*M*. *abscessus* ^nsGPL(+)^) on the cell surface are referred to as the smooth morphotype whereas those lacking nsGPL (*M*. *abscessus* ^nsGPL(-)^) are known as rough morphotype. *M*. *abscessus* smooth was obtained from ATCC (#19977) and *M*. *abscessus* rough was a clinical isolate cultured from a patient with cystic fibrosis (CF31) (Dr. Lindsay Caverly, National Jewish Health, Denver, Colorado).

### Bacterial cultures

Laboratory strains of *Escherichia coli* and *Salmonella* species were cultured in Luria Bertani (LB) broth to obtain stock cultures. All NTM strains were cultured in 7H9 broth with ADC enrichment supplement to obtain stock cultures. *Mtb* H37Rv was handled under BSL3 safety conditions as previously described [41].

### Synthetic bioactive LL-37

The synthetic human LL-37 peptide (NH_2_-LLGDFFRKSKEKIGKEFKRIVQRIKDFLRNLVPRTES-COOH) and scrambled LL-37 (NH_2_—GLKLRFEFSKIKGEFLKTPEVRFRDIKLKDNRISVQR—COOH) were synthesized by the Peptide Core, University of Colorado Anschutz Medical Campus. The peptides were purified by preparative reverse-phase high performance liquid chromatography (HPLC), verified by analytical reversed-phase HPLC, and molecular mass determined by electrospray mass spectrometry. Purity for both LL-37 and scrambled LL-37 peptide was >98%. The lyophilized peptides were stored at -20C and resuspended in 0.1% trifluoroacetic acid (final pH 2.0) before use.

### Antibacterial assays and bacterial detection

The antibacterial assay culture medium for LL-37 was previously optimized (RPMI-1640 supplemented with sodium bicarbonate pH 7.3; diluted 1:4 in distilled water) [21] and referred henceforth as the “LL-37 medium.” In each kill assay, 2x10^5^ to 2x10^6^ bacteria per 250 μl LL-37 medium (pH 7.0) were incubated with various concentrations of LL-37. The tubes were vigorously vortexed, rotated, and incubated at 37°C up to 96 hours. Killing efficacy was analyzed by performing serial dilutions of bacterial-peptide cocktail. Each dilution was plated in duplicate onto agar plates that were incubated at 37°C. *E*. *coli* and *Salmonella species* were inoculated on LB plates and incubated overnight, while rapidly-growing (*M*. *abscessus*) and slow-growing mycobacteria *(M*. *avium and M*. *intracellulare)* were incubated for 3–5 days or 10–14 days, respectively on 7H10 agar plates. Colony forming units (CFU) were manually scored. A minimum of three or more independent experiments were completed for all antibacterial assays.

### GPL isolation and thin layer chromatography

The presence of GPL from *M*. *avium* ^ssGPL^ and the absence of GPL from the *M*. *avium* ^ΔssGPL^ mutant were previously confirmed by thin layer chromatography (TLC) [40]. *M*. *abscessus* ^nsGPL(+)^ and *M*. *abscessus* ^nsGPL(-)^ variants were grown in separate 25 ml 7H9 broth cultures and GPL extracted using previously described methods [42, 43]. Lipids were saponified by incubation at 37°C for 20 minutes in 0.2M NaOH/methanol and neutralized with glacial acetic acid (0.1M final concentration). The samples were centrifuged at 20,000 x *g* for 10 minutes, evaporated using N_2,_ and resuspended in chloroform:methanol (2:1). A portion of the clarified organic phase was spotted onto silica plates for analysis by TLC using a chloroform:methanol:H_2_O (100:14:0.8) solvent system. Each plate was dipped in 0.1% orcinol/5%H_2_SO_4_ and placed under dry heat (100°C for 20 minutes) for visualization of lipids.

### Fraction purification from *M*. *abscessus* and *M*. *intracellulare*

Total lipid fractions were extracted from cell pastes of *M*. *abscessus* ^nsGPL(+)^ and *M*. *intracellulare* (11.4 g and 9.5 g, respectively). Briefly, pulses from a microfluidizer, intermittent cycles of sonication, and three to four cycles of bead beating (Zironia/Silica beads 11079101z BioSpec) were used to lyse the bacteria. Beads and insoluble debris were removed by centrifugation for five minutes at 3000 x *g* at 4°C. A portion of the resultant whole cell lysate (WCL) was reserved, with the remaining WCL further separated to generate cell wall (CW), cell membrane (CM), and cytosol fractions by established methods [44, 45]. Lipids of each subcellular fraction were obtained by sequential extraction with chloroform:methanol (2:1) as described previously [45]. Finally, the insoluble cell wall (ICW) fraction was obtained by exhaustive extraction of delipidated CW with SDS to remove proteins followed by harvest of ICW via centrifugation [46]. Each fraction was aliquoted into pre-weighed vials, lyophilized under N_2_ air, and re-weighed to determine component mass.

### *E*. *coli* bioassays as a readout for LL-37 antimicrobial activity

To test whether LL-37 exposed to NTM- or NTM-derived fractions is inactivated, *E*. *coli* growth was monitored by two different bioassays. In the first bioassay, NTM cultures incubated with LL-37 for 96 hours were centrifuged at 6,000 *x g*, which pellets the bacteria, and leaves any residual LL-37 in the supernatant. Next, the culture supernatant was transferred into new microfuge tubes to which 2x10^5^ *E*. *coli* was inoculated and incubated for four hours. After incubation, serial dilutions were performed and inoculated onto duplicate LB agar plates, incubated for four hours, and *E*. *coli* CFU enumerated. In the second bioassay, 10 μg of each NTM-derived lipid fraction was resuspended in DMSO to which 250 μl of LL-37 medium was added. The final concentration of DMSO in each sample was <0.1%. Next, 25μg/ml of fresh LL-37 was added to each NTM fraction and pre-incubated at 37°C for four hours. Subsequently, 2x10^5^ *E*. *coli* was inoculated into each of the samples, incubated for an additional 16 hours, and swabbed onto LB agar plates to assess bacterial growth.

### Statistical analyses

The data were analyzed with GraphPad using paired *t*-tests or two-way ANOVA to determine statistical significance. Values with p<0.05 were considered statistically significant. Data are expressed as means ± S.E.M. for three or more independent determinations for each experimental point.

## Results

### Synthetic LL-37 possesses broad-spectrum antibacterial activity

**Fig 1:**
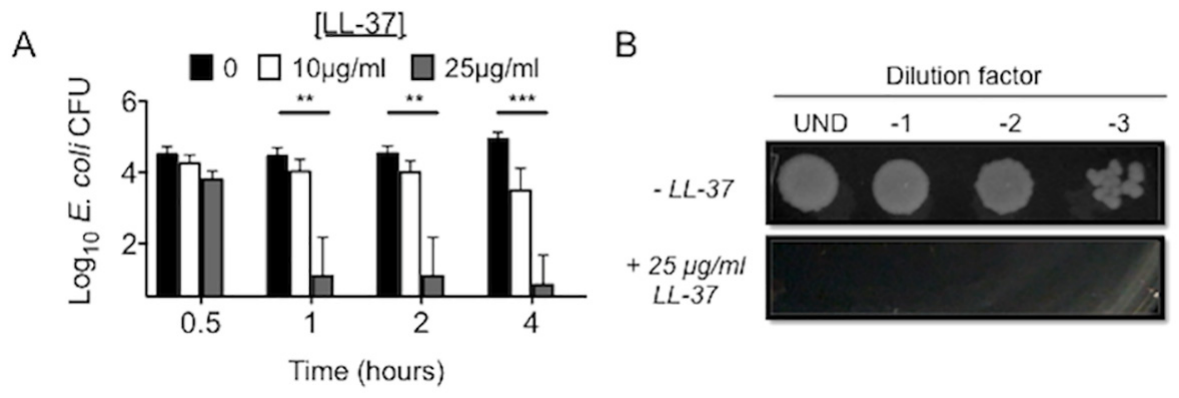
*E*. *coli* is susceptible to LL-37. (A) Log_10_ CFU of *E*. *coli* after 4 hours of incubation with 0, 10, or 25 μg/ml of LL-37. **p< 0.001; ***p<0.0001. Data are the mean ± SEM of 6 independent experiments. (B) Images were taken of each serial dilution on LB agar from *E*. *coli* cultures incubated for 4 hours in the absence or presence of 25 μg/ml of LL-37.

**Fig 2:**
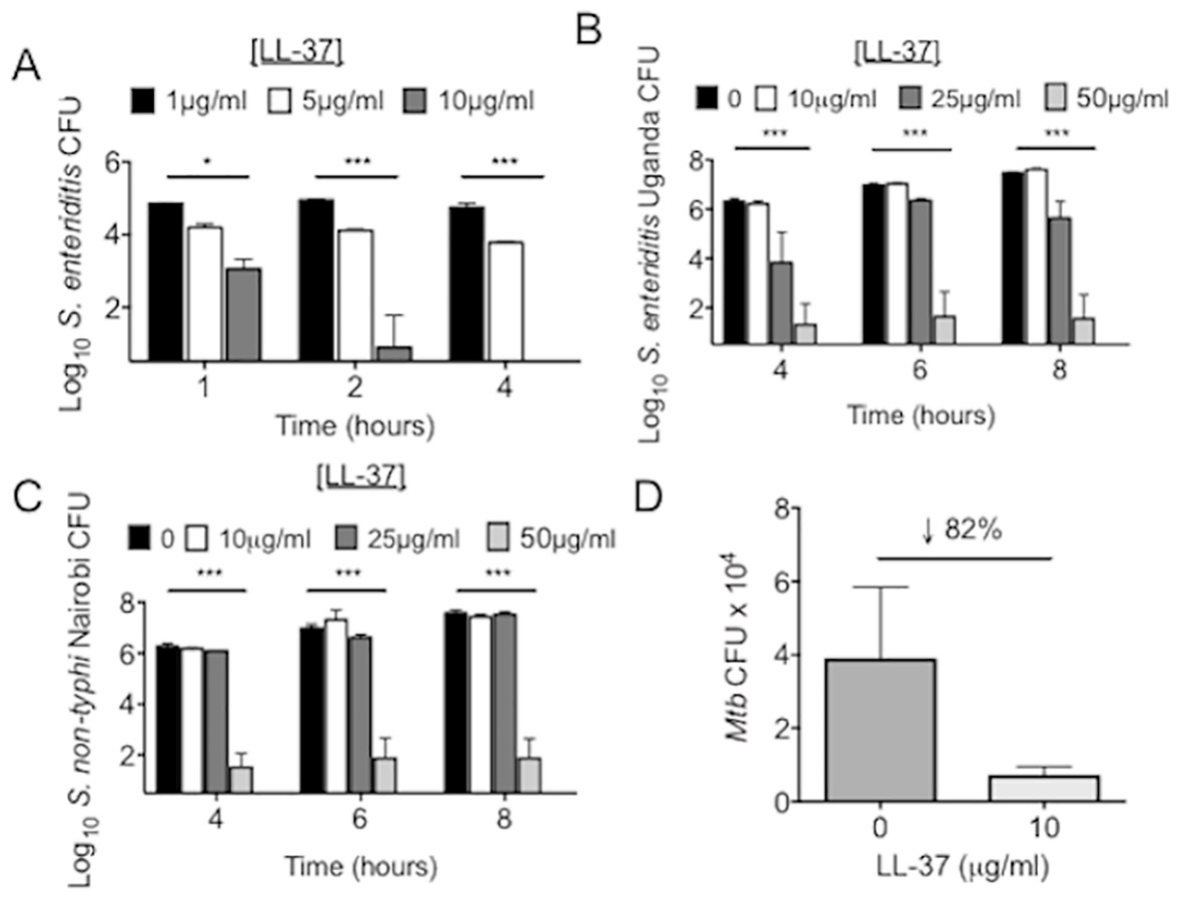
LL-37 demonstrates broad-spectrum antimicrobial activity. Log_10_ CFU after 1–8 hours of incubation of a (A) laboratory isolate of *Salmonella enteriditis* or clinical isolates of (B) *Salmonella enteriditis* (Uganda) or (C) *Salmonella non-typhi* (Nairobi) with 0–50 μg/ml of LL-37. *p< 0.01; **p< 0.001; ***p<0.0001. Data are the mean ± SEM of 3–6 independent experiments. (D) *Mtb* H37Rv were incubated with 10 μg/ml LL-37 and the percent change in CFU calculated after 96 hours incubation. n = 3 independent experiments.

To validate the antibacterial activity of the synthesized LL-37, LL-37 at concentrations of 0–25 μg/mL was added to *E*. *coli* cultures and CFU quantified. Incubation with 25 μg/ml LL-37 effectively killed *E*. *coli* after four hours (Fig 1A and 1B), consistent with previous reports [47, 48]. LL-37 at 1–50 μg/ml also demonstrated antimicrobial activity against various species of *Salmonella* including a non-pathogenic laboratory isolate (Fig 2A) and two pathogenic clinical isolates (Fig 2B and 2C). Similar to a previous report [21], ten μg/ml of LL-37 incubated with *Mtb* reduced viability, but the reduction was not statistically significant (Fig 2D). Overall, synthetic LL-37 has antimicrobial activity against a variety of bacteria.

### *M*. *avium* Chester and *M*. *intracellulare* are resistant to LL-37

**Fig 3:**
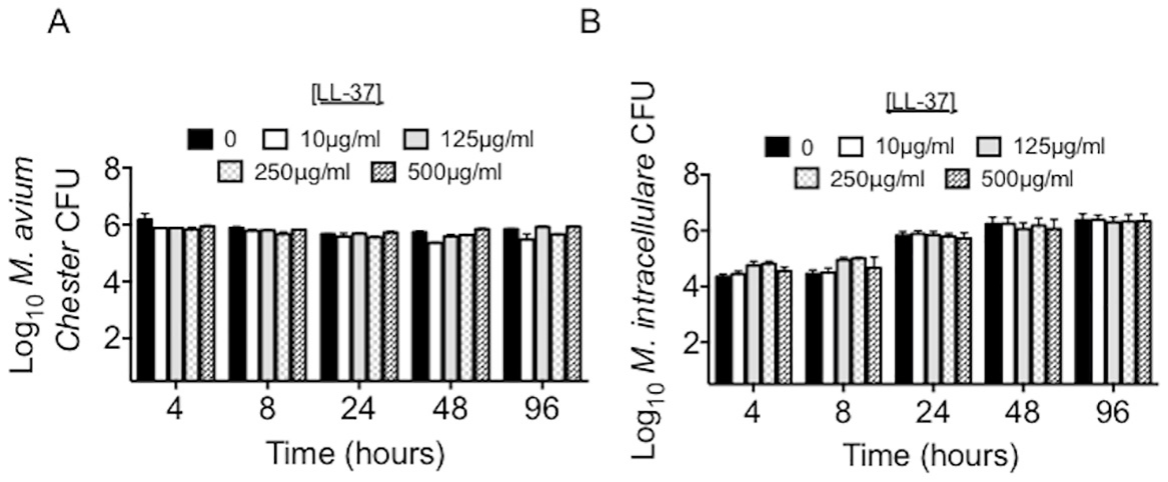
Pathogenic *M*. *avium* and *M*. *intracellulare* are resistant to LL-37. Growth curves of (A) *M*. *avium* Chester (p = 0.28) and (B) *M*. *intracellulare* (p = 0.89) after incubation with 0, 10, 125, 250, or 500 μg/ml of LL-37. Data are the mean ± SEM of 3–6 independent experiments.

To evaluate the effectiveness of LL-37 against NTM species, *M*. *avium* Chester and *M*. *intracellulare* were separately incubated with 10, 125, 250, and 500 μg/ml LL-37 for up to 96 hours and CFU determined. In contrast to the aforementioned findings, there was no significant change in the viability of *M*. *avium* Chester or *M*. *intracellulare* at all LL-37 concentrations and time points tested (Fig 3A and 3B). We also found no significant change in viability of 48-hour log phase cultures incubated with up to 250 μg/ml of LL-37 (data not shown).

### *M*. *avium* ^ssGPL^ and *M*. *avium* ^ΔssGPL^ serovar 8 are equally resistant to LL-37

**Fig 4:**
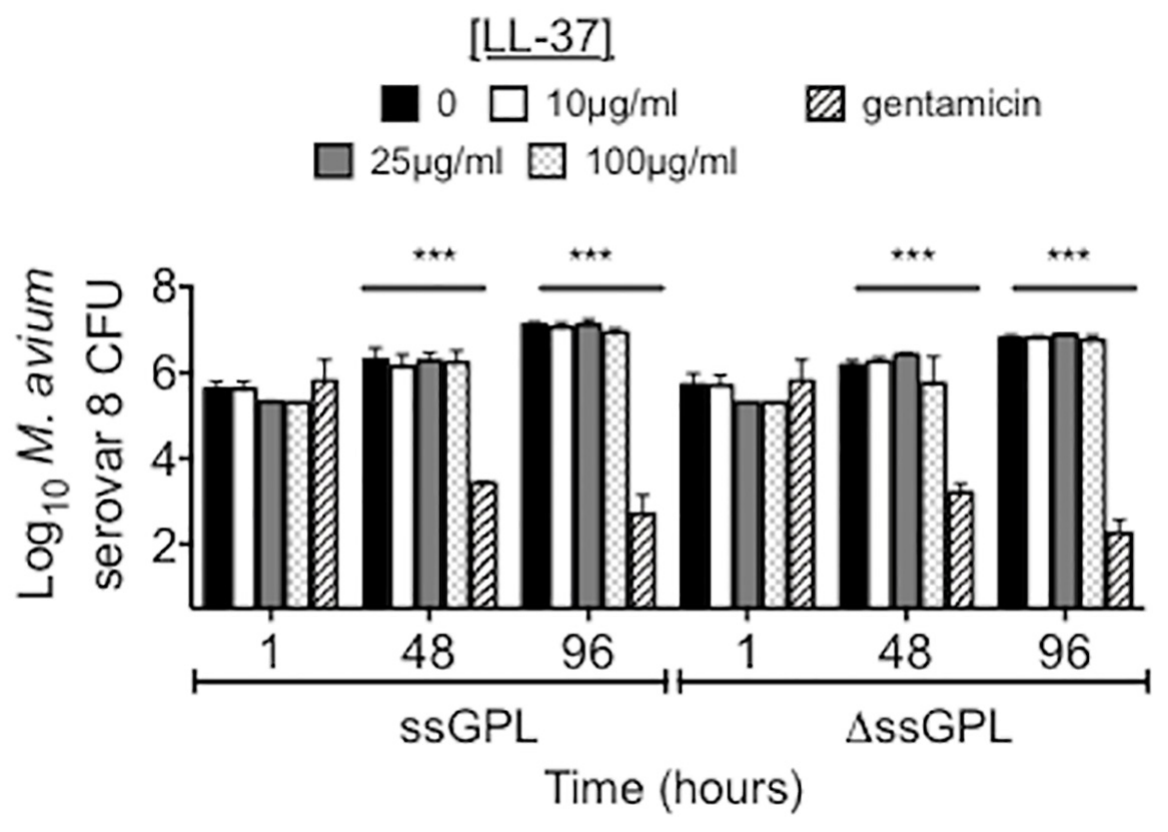
ssGPL does not contribute to the resistance of *M*. *avium* to LL-37. CFU determination of *M*. *avium* ^ssGPL^ and *M*. *avium* ^ΔssGPL^ serovar 8 after incubation with 0, 10, 25, and 100 μg/ml LL-37 and 20 μg/ml gentamicin. ***p<0.0001. Data are the mean ± SEM of 3 independent experiments.

To investigate whether ssGPL contribute to the resistance of *M*. *avium* to LL-37, *M*. *avium* ^ssGPL^ serovar 8 and *M*. *avium* ^ΔssGPL^ were incubated with increasing concentrations of LL-37 and CFU quantified at various times. As shown in Fig 4, both *M*. *avium* ^ssGPL^ and *M*. *avium* ^ΔssGPL^ were resistant to LL-37 at all concentrations and time points examined. In contrast, gentamicin significantly reduced the growth of both variants after 48 and 96 hours.

### Both *M*. *abscessus* ^*nsGPL(+)*^ *and* ^*nsGPL(-)*^ variants are equally resistant to LL-37

**Fig 5:**
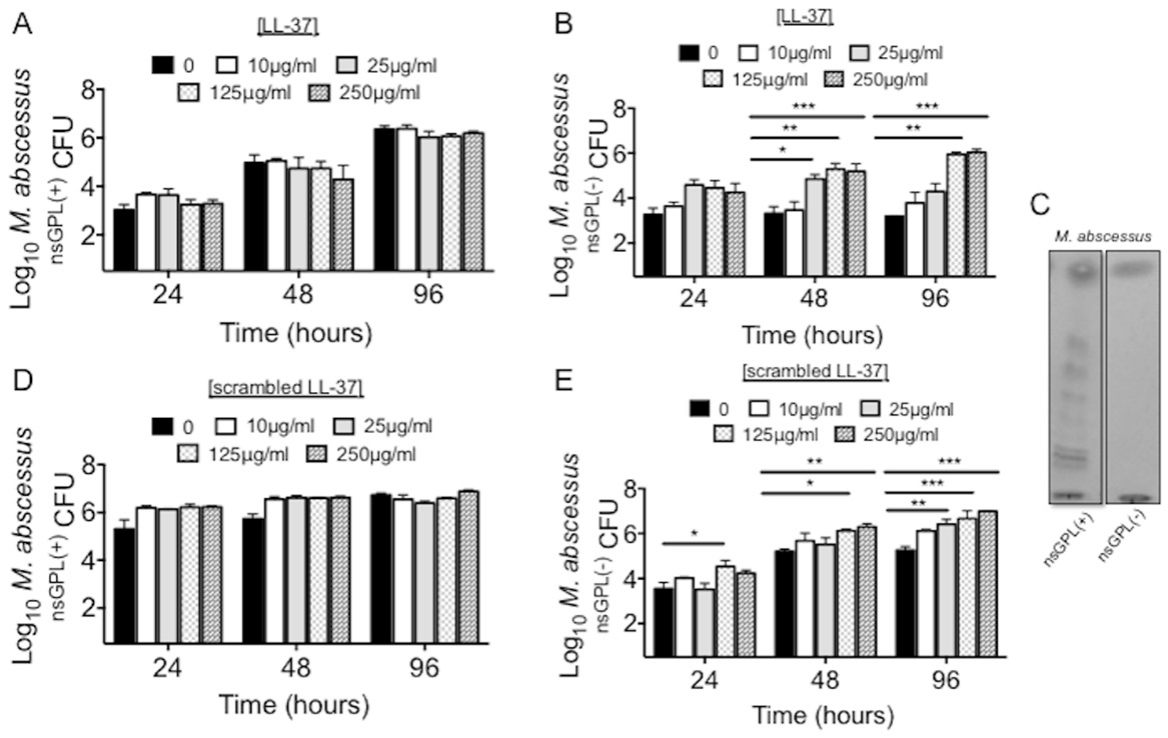
nsGPL do not mediate the resistance of *M*. *abscessus* to LL-37. (A-B) CFU determination of *M*. *abscessus* ^nsGPL(+)^ in the presence of the indicated concentrations of native LL-37 (p = 0.69). Data are the mean ± SEM of 4 independent experiments. (B) CFU determination of *M*. *abscessus* ^nsGPL(-)^ in the presence of the indicated concentrations of native LL-37. Data are the mean ± SEM of 4 independent experiments. *p<0.01; **p< 0.001; ***p<0.0001. (C) Thin-layer chromatography demonstrates the presence and absence of GPL in *M*. *abscessus* ^nsGPL(+)^ and *M*. *abscessus* ^nsGPL(-)^, respectively. (D-E) CFU determination of *M*. *abscessus* ^nsGPL(+)^ and *M*. *abscessus* ^nsGPL(-)^, respectively in the presence of the indicated concentrations of scrambled LL-37 peptide. Data are the mean ± SEM of 3 independent experiments. *p<0.01; **p< 0.001; ***p<0.0001.

We also incubated a strain of *M*. *abscessus* that contains nsGPL (*M*. *abscessus* ^nsGPL(+)^) with various concentrations of LL-37 for up to 96 hours. We found that *M*. *abscessus* ^nsGPL(+)^ was completely resistant to LL-37 at all concentrations and time points tested (Fig 5A). To determine whether the nsGPL present on the wildtype *M*. *abscessus* is responsible for this resistance, the growth of a *M*. *abscessus* variant devoid of nsGPL (*M*. *abscessus* ^nsGPL(-)^) was tested against LL-37. After 48 and 96 hours of incubation in the absence of LL-37, the growth of *M*. *abscessus* ^nsGPL(-)^ was significantly slower than *M*. *abscessus* ^nsGPL(+)^ (Fig 5B compared to 5A, black bars). In the presence of LL-37, neither *M*. *abscessus* strain was killed by LL-37. On the contrary, *M*. *abscessus* ^nsGPL(-)^ was not only resistant to LL-37, but unexpectedly showed significant increases in growth when incubated with increasing concentrations of LL-37 compared to the untreated cultures (Fig 5B). Thin layer chromatography confirmed the presence and absence of GPL in *M*. *abscessus* ^nsGPL(+)^ and *M*. *abscessus* ^nsGPL(-)^, respectively (Fig 5C).

To examine whether the enhanced growth of the *M*. *abscessus* ^nsGPL(-)^ cultured with LL-37 was dependent on the primary structure of LL-37, *M*. *abscessus* ^nsGPL(-)^ and *M*. *abscessus* ^nsGPL(+)^ strains were incubated for 24, 48, and 96 hours with varying concentrations of an LL-37 peptide in which the amino acid sequence are randomly scrambled and CFU determined. Similar to that seen following incubation with LL-37, *M*. *abscessus* ^nsGPL(+)^ viability was not affected by scrambled LL-37 at any concentration or time point tested (Fig 5D) whereas *M*. *abscessus* ^nsGPL(-)^ growth also increased after incubation with scrambled LL-37 (Fig 5E).

### LL-37 exposed to NTM or NTM-derived fractions loses antibacterial activity

**Fig 6:**
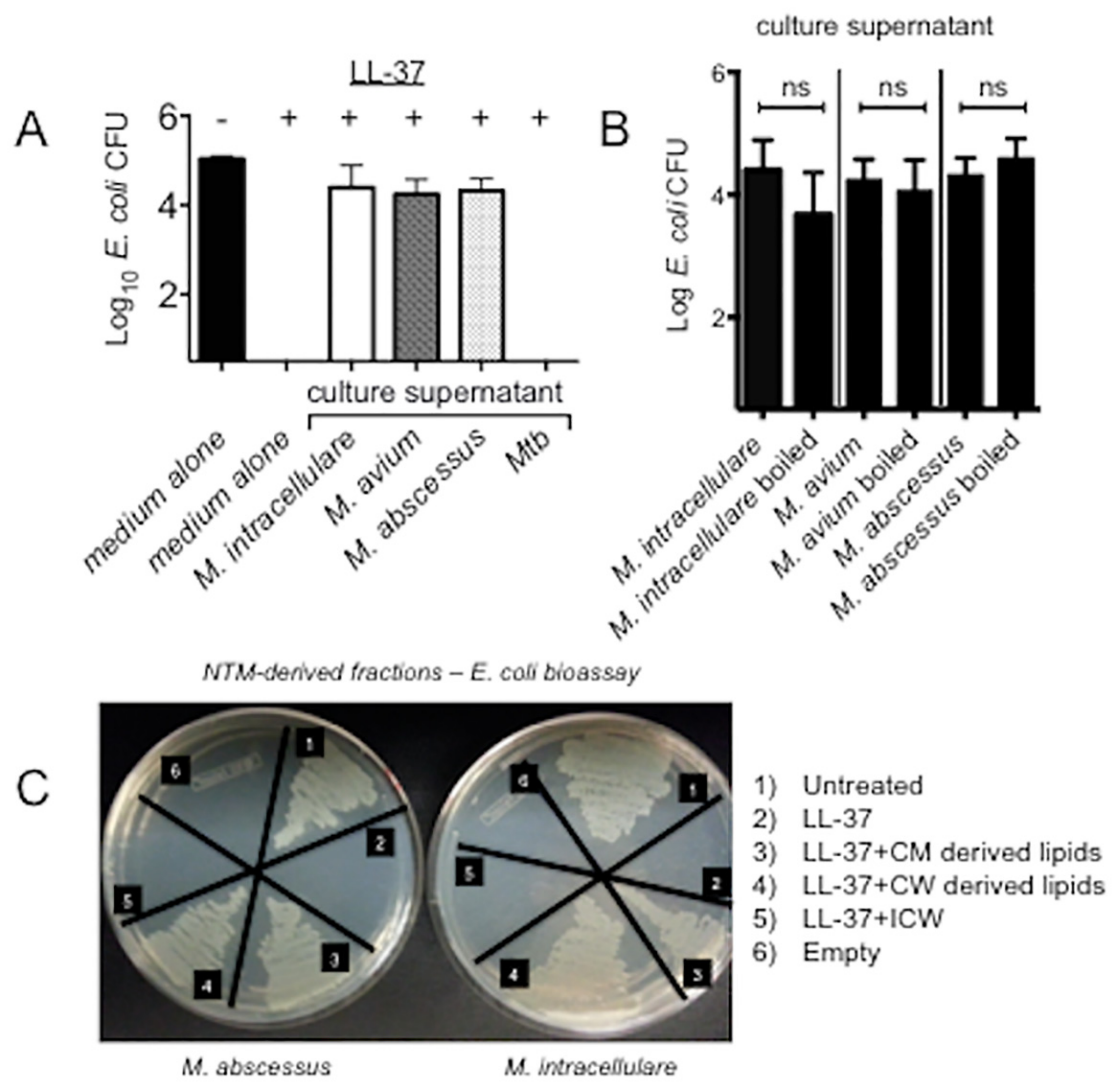
Loss of LL-37 activity after exposure to NTM or NTM-derived lipids. *E*. *coli bioassays* were used to evaluate LL-37 activity. (A) LL-37 remaining in NTM (but not *Mtb*) culture supernatant no longer kills *E*. *coli*. (B) *E*. *coli* survives in untreated or boiled NTM culture supernatants to which fresh LL-37 was added. (C) *E*. *coli* survival following incubation with *M*. *abscessus* or *M*. *intracellulare* derived cell fractions. CM = cell membrane, CW = cell wall, ICW = insoluble cell wall fraction.

Since we did not observe killing of NTM by LL-37, we investigated the possibility that NTM-exposed LL-37 may have become inactivated. In a bioassay, *E*. *coli* was added to culture supernatants of *M*. *intracellulare*, *M*. *avium* Chester, *M*. *abscessus*, and *Mtb* originally incubated with 500 μg/ml LL-37 for 96 hours. *E*. *coli* cultured in medium alone showed viable cells, which were killed in the presence of LL-37 (Fig 6A). However, when *E*. *coli* was incubated with LL-37-containing NTM culture supernatants, no significant decrease in viable *E*. *coli* was observed (Fig 6A); in contrast, no viable *E*. *coli* was recovered after incubation with LL-37-containing *Mtb* culture supernatant (Fig 6A).

To determine if the NTM component(s) that inactivates LL-37 is heat labile, culture supernatants from *M*. *intracellulare*, *M*. *avium* Chester, and *M*. *abscessus* incubated for 96-hours without the addition of LL-37 were boiled and cooled to room temperature prior to the addition of *E*. *coli* and fresh LL-37 peptide. These bioassays demonstrated that *E*. *coli* remained viable and replicated well despite incubation with LL-37 in the presence of boiled NTM culture supernatant (Fig 6B).

Based on our findings thus far, it appears that a secreted, or surface exposed, heat-stable component of the NTM cell envelope inactivates LL-37. To begin to dissect which component(s) of NTM inactivates LL-37, large-scale preparations of *M*. *abscessus* and *M*. *intracellulare* were prepared and chloroform:methanol was used to separate the total lipids of the CM and CW fractions from the glycans and proteins. Fresh LL-37 (25 μg/ml) and *E*. *coli* were added to the total lipid fractions of the CM/CW or the ICW fraction per unit of dry weight to test the ability of each fraction to inactivate LL-37. *E*. *coli* was killed when incubated with LL-37 alone and LL-37 with ICW; however, viable *E*. *coli* was recovered after incubation with LL-37 plus either *M*. *abscessus* or *M*. *intracellulare* CM or CW total lipid fractions (Fig 6C). As a control, *E*. *coli* was incubated with just the buffer solutions used to resuspend each of the *M*. *abscessus* and *M*. *intracellulare* fractions (vehicle control); in all cases, *E*. *coli* replicated in the absence of LL-37, but were killed in the presence of LL-37 in these NTM-naïve solutions (data not shown).

## Discussion

We discovered that LL-37, despite being highly active against *E*. *coli*, various *Salmonella* species, and *Mtb H37Rv*, was inactive against four NTM species tested. *M*. *avium* and *M*. *abscessus* strains with genetic disruption for their respective GPLs (*M*. *avium* ^ΔssGPL^ and *M*. *abscessus* ^*nsGPL*(-)^) remained resistant to LL-37. Thus, GPL is unlikely to be the NTM component that mediates resistance to LL-37. Indeed, the growth of *M*. *abscessus* ^nsGPL(-)^ unexpectedly and consistently increased when incubated with either native LL-37 or scrambled peptide. Our data also indicate that LL-37 exposed to either NTM or NTM-derived CM or CW total lipid fractions becomes inactivated. Taken together, our findings indicate that NTM are resistant to LL-37, inactivate the antimicrobial activity of LL-37, and that the NTM-derived component(s) that inactivate LL-37 are likely to be polar lipids or similarly hydrophilic component(s) of the NTM cell envelope that is extractable with an organic solvent.

The finding that many species of NTM are resistant to LL-37 is consistent with a recent report demonstrating resistance of *M*. *avium hominissuis* to LL-37 [22]. Although LL-37 was the only antimicrobial peptide tested in our studies, resistance to antimicrobial peptides may be a global defense mechanism of NTM. The differential activity of LL-37 against NTM and *Mtb* is interesting, but not surprising considering they contain different cell wall components; *e*.*g*., compared to lipoarabinomannan of NTM, the mannose-capped lipoarabinomannan of *Mtb* is distinct in structure and elicits a different host-immune response [49]. Moreover, it is well established that the surface-exposed lipids of the different mycobacterial species are highly varied in composition, location, and arrangement which may impact susceptibility or resistance to antimicrobial peptides [24]. *Mtb* is a strict intracellular organism and does not survive for extended periods outside of the host. Compared to *Mtb*, the cell envelope of NTM is likely to be considerably more impermeable to enhance survival in environments that vary greatly; *e*.*g*., natural and made-made water systems, soil, and biofilms that can vary with extremes of temperature, humidity, and nutrients [50]. A global comparative lipidomics approach, using recently established methods and mycobacterial lipid databases [51–53] will be required to identify lipids unique to NTM that possesses this activity.

Although our findings would suggest that GPLs—whether ssGPL in *M*. *avium* or nsGPL in *M*. *abscessus*—do not inactivate LL-37, there are at least 31 other serovar-specific *M*. *avium* GPL not examined in this study [36]. But based on the available evidence, it is reasonable to suspect NTM GPL do not mediate resistance to LL-37. In future studies, it would be informative to determine whether purified ssGPL from the different *M*. *avium* species including *M*. *avium hominissuis*, modulate the structure of LL-37.

The finding that the growth of the *M*. *abscessus* ^*nsGPL*(-)^ variant increased with increasing concentrations of LL-37 (or scrambled LL-37) was unexpected. One interpretation of these findings is that the experimental conditions alone were unfavorable for the *M*. *abscessus* ^*nsGPL*(-)^ variant, but adding increasing concentrations of LL-37 may have sufficiently supplemented the conditions to facilitate growth. The exact mechanisms to explain this observation are unknown, but may be due to increased activity of uptake transporters or permeases that internalize and metabolize LL-37 [54, 55]. We recognize an important limitation to these studies is that the *M*. *abscessus* ^*nsGPL*(-)^ and *M*. *abscessus* ^*nsGPL*(+)^ variants were not derived from the same parental *M*. *abscessus* strain; thus, we cannot exclude the possibility that the observed increase in the growth of the *M*. *abscessus* ^*nsGPL*(-)^ variant is due to attributes unique to this particular variant which may be independent of the absence of nsGPL. Isogenic *M*. *abscessus* ^*nsGPL*(-)^ and *M*. *abscessus* ^*nsGPL(+)*^ variants should be used in future studies to confirm these findings.

Bacterial evasion of host-protective LL-37 is common. In fact, there are a wide variety of evasion strategies employed by numerous bacterial species to avoid LL-37 including modification of normally anionic cell surface constituents with cationic molecules to repel LL-37, antimicrobial peptide trapping mechanisms, secretion of proteases that cleave LL-37, and downregulation of antimicrobial peptide transcription (reviewed in [50]). Our findings indicate that components of the NTM cell envelope contribute to LL-37 resistance, and, more specifically, heat-stable CM and/or CW lipids exert the anti-LL-37 activity.

In summary, the finding that clinically relevant species of NTM are uniquely resistant to and neutralize LL-37 provides a pathogenic mechanism for why it is difficult to eradicate these organisms once the infection becomes established. Elucidating the mechanism by which NTM resist and inactivate LL-37 will provide leverage in developing more unconventional approaches to therapeutics with novel mechanisms of action to slow the progression of this emerging lung disease; *i*.*e*., development of agents to counter the NTM component(s) that neutralize LL-37.
